# Potential of Salt Tolerant PGPR in Growth and Yield Augmentation of Wheat (*Triticum aestivum* L.) Under Saline Conditions

**DOI:** 10.3389/fmicb.2020.02019

**Published:** 2020-10-02

**Authors:** Aniqa Nawaz, Muhammad Shahbaz, Asadullah, Asma Imran, Muhammad U. Marghoob, Muhammad Imtiaz, Fathia Mubeen

**Affiliations:** ^1^Stress Physiology Lab, Department of Botany, University of Agriculture, Faisalabad, Pakistan; ^2^Microbial Physiology Lab, Soil and Environmental Biotechnology Division, National Institute for Biotechnology and Genetic Engineering, Constituent College of Pakistan Institute of Engineering and Applied Sciences, Islamabad, Pakistan; ^3^Phytohormone Lab, Department of Plant Sciences, Quaid-I-Azam University, Islamabad, Pakistan

**Keywords:** plant growth promoting rhizobacteria, salt tolerance, osmotically active compounds, salt stress, climate change

## Abstract

Soil salinity has emerged as a major obstacle to meet world food demands. Halo-tolerant plant growth promoting rhizobacteria (PGPR) are potential bioinoculants to enhance crop productivity in saline agriculture. Current work was aimed at studying individual or synergetic impact of salt tolerant PGPR on wheat growth and yield under saline conditions. A pot experiment was conducted on two wheat genotypes (Aas-11; salt tolerant and Galaxy-13; salt sensitive) inoculated with *Pseudomonas fluorescence, Bacillus pumilus*, and *Exiguobacterium aurantiacum* alone and in consortium. The salt tolerant variety (Aas-11) exhibited maximum root fresh (665.2%) and dry biomass (865%), free proline (138.12%) and total soluble proteins (155.9%) contents, CAT (41.7%) activity and shoot potassium uptake (81.08%) upon inoculation with *B. pumilus*, while improved shoot dry weight (70.39%), water (23.49%) and osmotic (29.65%) potential, POD (60.51%) activity, enhanced root potassium (286.36%) and shoot calcium (400%) were manifested by *E. aurantiacum.* Highest shoot length (14.38%), fresh weight (72.73%), potassium (29.7%) and calcium (400%) acquisition as well as glycinebetaine (270.31%) content were found in plants treated with PGPR consortium. On the other hand, in the salt sensitive variety (Galaxy-13), *P. fluorescens* treated plants showed significantly improved leaf-water relations, glycinebetaine (10.78%) content, shoot potassium (23.07%), root calcium (50%) uptake, and yield parameters, respectively. Plant root length (71.72%) and potassium content (113.39%), root and shoot fresh and dry biomass, turgor potential (231.02%) and free proline (317.2%) content were maximum upon PGPR inoculation in consortium. Overall, Aas-11 (salt tolerant variety) showed significantly better performance than Galaxy-13 (salt sensitive variety). This study recommends *B. pumilus* and *E. aurantiacum* for the salt tolerant (Aas-11) and *P. fluorescens* for the salt sensitive (Galaxy-13) varieties, as potential bioinoculants to augment their growth and yield through modulation of morpho-physiological and biochemical attributes under saline conditions.

## Introduction

Climate change, a hot topic of the current era, has affected planet earth in different ways and a rapid increase in saline landscapes is one of them that ultimately leads to global food insecurity and reduced agricultural productivity (Bharti et al., 2016). Around the globe, 20% of irrigated land is severely damaged by salt accumulation (Selvakumar et al., 2014). This land deterioration is expected to reach up to 50% by the year 2050 (Hossain, 2019). Almost 70% yield loss has been reported among cereal crops including wheat, rice, maize, and barley due to soil contamination by salinity and sodicity (Rajendran et al., 2009; Hussain et al., 2019).

So far, reclamation of such soils is being done by utilizing a variety of inorganic (gypsum, limestone, sulfuric acid and derivatives of sulfur, synthetic fertilizers), and organic (green and farm yard manure, industrial waste like press mud) measures (Qayyum et al., 2016). Similarly, Plant breeders and biotechnologists are in a constant struggle for the development of salt tolerant crop varieties either through natural selection, QTL mapping, marker assisted selection or by genetic manipulation via introduction of salt tolerant genes obtained from other organisms (Qadir et al., 2017). However, at field level, due to multiple factors, satisfactory outcomes have not been observed by such biological means for stress tolerance enhancement among the agro-economical significant crops (Khare et al., 2018).

Recently, exploitation of root adhering plant growth promoting rhizobacteria (PGPR) inhabiting hyper- saline conditions has gained attention as an alternative eco-friendly biological approach to get better crop productivity from salt deteriorating lands (Talaat, 2015). Improvement in plant growth aided by these microbes is well documented (Barnawal et al., 2012; Bharti et al., 2014). These halophilic/halotolerant plant growth-promoting rhizobacteria employ their key mechanisms by colonizing the plant rhizosphere to combat brutal environmental stresses and subsequent ruinous yield penalties. Strategies adopted by these microbes include *de novo* synthesis of osmolytes for cellular osmotic adjustment, regulation of ionic transporters and maintenance of homeostasis to reduce toxic effects of Na^+^ and Cl^–^ ions, activation of reactive oxygen species scavenging defense system of plants to cope with deleterious effects of oxidative stress, respectively (Munns and Tester, 2008). Moreover, these microbes synthesize phytohormones, ACC deaminase, biological nitrogen fixation, siderophores, exopolysaccharides, volatile compounds and antifungal or antibacterial metabolites, mobilization of mineral ions, enhancement of photosynthesis, osmotic adjustment through accumulation of osmotically active metabolites like amino acids, sugars, polyols and betaines and detoxification of reactive oxygen species by antioxidants (Talaat and Shawky, 2013; Shabani and Sabzalian, 2016). Hence, these tiny creatures, by using different direct and indirect mechanisms, support plants to combat many biotic and abiotic challenges (Talaat, 2015; Subramanian et al., 2016).

By simple definition, salinity is a form of chemical (abiotic) factor that causes accumulation of soluble salts in the rhizospheric system. This condition adversely affects plant metabolism in two ways. Initially, high concentration of salts induces hyperosmotic and hyperionic situations which damage root architecture consequently leading to impaired water and nutritional acquisition. This eventually triggers secondary stress, i.e., oxidative stress, ultimately resulting in denaturation of DNA and proteins, and membrane instability due to lipid peroxidation. All these phenomena lead to programmed cell death and the collapse of the entire plant (Meloni et al., 2003; Kim et al., 2014).

Wheat (*Triticum aestivum* L.) is a staple food for 35% of the human population (Agriculture statistics of Pakistan 2017–19). Different plant species have acquired various ranges of stress tolerance. Some wheat varieties, for example, can sustain up to 10 dS/cm salinity level with minor yield losses and fall under the salt tolerant category (Munns et al., 2006). However, there is an immense need to enhance crop productivity up to 57% to meet ever increasing food demands by the year 2050 in parallel with continuous 1 to 2% land loss caused by salinity per year (Hossain, 2019).

It was hypothesized that PGPR residing in hyper-saline ecological conditions have the potential to modulate plant physiology by induced systemic tolerance to promote growth in salt degraded lands. Therefore, the present study was designed to evaluate the role of halotolerant PGP microbes by using two commonly used strategies (single strains and consortium) on two contrasting genotypes of wheat plant (salt tolerant and salt susceptible) and parameters used to investigate their role in plant growth and yield improvement include morpho-physiological and biochemical characteristics. This study will be helpful to explore the potential of native salt tolerant strains of PGPR and further their utilization as biofertilizer for wheat crop to minimize yield losses due to salt stress. In the future, this could lead to developing an effective bioformulation for such problematic soils.

## Materials and Methods

### Experimental Area and Materials

This experimental work was conducted in the greenhouse, located at University of Agriculture, Faisalabad, Pakistan during wheat growing season November 2015–April 2016. Seeds of wheat (salt tolerant variety; Aas-11 and salt susceptible variety; Galaxy-13) were obtained from Ayub Agriculture Research Institute, Faisalabad, Pakistan. Three pre-characterized halotolerant PGPR strains, *Pseudomonas fluorescens* (Accession # KX644132), *Bacillus pumilus* (Accession # KX580768) and *Exiguobacterium aurantiacum* (Accession # KX580769) were collected from NBRC, Microbial Physiology Lab, SEBD, NIBGE, Faisalabad. The salt tolerance profiling on the basis of minimal inhibitory concentration and PGP characteristics (phosphate solubilization, IAA production and ACC metabolism) of these strains were assessed (qualitatively and quantitatively) based on selective media in a previous study (Ullah, 2019). Ullah and Bano (2019) demonstrated that these PGPR have significantly improved the growth and yield of maize grown in saline sodic soil as well physico-chemical properties of soil. Compatibility of these strains was also assessed using a cross streak method (Semenov et al., 2007) on NaCl supplemented LB-medium prior to seed inoculation. Pure saline soil used for the experiment was brought from field area of Biosaline Research Station, Pakka Anna, Faisalabad, Pakistan. The soil contained EC_e_: 13.41, pH: 9.1, organic matter: 1.39%, available nitrogen: 1.4 mg kg^–1^, available phosphorus: 19.6 mg kg^–1^, extractable potassium: 2.1 mg kg^–1^, sodium: 55 mg kg^–1^, chloride: 999.96 mg kg^–1^ and soil texture was clay loam.

### Seed Sterilization and Inoculation

Seeds were surface sterilized with 10% sodium hypochlorite solution and subsequent washing with autoclaved distilled water prior to inoculation with PGPR (Cheng et al., 1997). Inocula were prepared by transferring an 24 h old bacterial colony into Luria Bertani broth, kept on shaker at 120 rpm overnight at 28°C, centrifuged to get pellets, re-suspended in distilled water and optical density (at 660 nm) was adjusted to be 1, which was equal to 10^–6^ cells/ml. Then, surface sterilized wheat seeds were soaked in bacterial inocula containing single PGPR strains, and the consortium of three bacterial cultures for 2 h, while for control treatment seeds were soaked in autoclaved distilled water, respectively. Seeds were sown in plastic pots each containing 8.5 kg sterilized soil. Sowing was done at the rate of 20 seeds pot^–1^ at the depth of 1.5 inch. Thinning was done at the two-leaf stage up to 10 plants per pot^–1^. Irrigation was carried out with tap water (pH 7) following sufficient intervals. The experiment was based on completely randomized factorial design comprised of five treatments for each variety with five replications (Table 1). Data was recorded 70 days after sowing at vegetative stage to evaluate the impact of PGPR on growth and physiochemical attributes. Yield data was collected at crop maturity level.

**TABLE 1:** Description of experimental components (treatments).

Notations	Treatments
T_1_	Seed soaked in autoclaves distilled water (control or uninoculated)
T_2_	Seeds inoculated with *P. fluorescens*
T_3_	Seeds inoculated with *B. pumilus*
T_4_	Seeds inoculated with *E. aurantiacum*
T_5_	Seeds inoculated with consortium (3 bacterial strains used in T_2_, T_3_, and T_4_)

### Morpho-Physiological and Biochemical Analysis of Plants

#### Morphological Parameters of Plant

Data regarding root and shoot length of randomly selected plants was recorded right after sampling by using a field meter rod. Fresh weight of root and shoot was measured by electric balance. Dry biomass of 7 days oven dried samples at 115°C was recorded using the same electric balance.

#### Physiological Parameters of Plant

##### Water relation attributes

###### Water potential

Leaf-water potential was measured according to the method of Scholander et al. (1964). For that purpose, a fully expanded young leaf was excised from each plant. At dawn leaf water potential was measured by using Scholander type pressure chamber (Arimad-2-Japan).

###### Osmotic potential

The same leaves were used for osmotic potential determination and stored in a freezer at −20°C for at least 7 days. After 7 days, the sap was extracted by pressing them with glass rod. The sap was placed on an osmometer [Wescor Vapor Pressure Osmometer (Model VAPRO 5520, El Cajon, CA, United States)] for the measurement of solute potential (Ball and Oosterhuis, 2005).

###### Turgor potential

Turgor potential was calculated as the difference between water potential and osmotic potential by following the equation as cited by Nobel (1999).

Ψp=Ψw-Ψs

### Biochemical Parameters of Plant

#### Estimation of Total Soluble Proteins

The soluble proteins of the samples were determined by the Bradford method (Bradford, 1976). To extract protein, 0.25 g fresh leaves were grinded using a tissue grinder in 5 ml of 50 mM cooled phosphate buffer (pH 7.8) placed in an ice bath. The homogenate was centrifuged at 15,000 rpm for 15 min at 4°C. The supernatant was used for protein determination. Each sample, 100 μl, was taken in an Eppendorf tube and mixed with 1.0 ml of Bradford reagent. These sample solutions were incubated at 37°C for 10–15 min along with the blank and absorbance was noted at 595 nm using a spectrophotometer (IRMECO U2020).

#### Determination of Antioxidant Enzymatic Activity

##### Enzyme extraction

Enzymatic antioxidants of wheat plants were extracted by grinding 0.25 g of fresh leaf material in 5 ml of 50 mM cooled potassium phosphate buffer (pH 7.8). This homogenized material was centrifuged at 10000 × *g* for 22 min at 4°C. The pellet was discarded and the supernatant was used for the estimation of activities of different antioxidants enzymes.

##### Superoxide dismutase (SOD)

The activity of superoxide dismutase (SOD) was determined by monitoring its potential to cause inhibition in the photo reduction of nitro blue tetrazolium chloride (NBT) by following the procedure given by Giannoplitis and Ries (1977). The reaction mixture (3 ml of 50 mM potassium phosphate buffer (pH 7.8), 75 mM MEDTA, 13 mM methionine, 50 μM NBT, riboflavin, 1.3 μM) and 50 μl enzyme extract for the detection of enzyme activity. The tubes with reaction mixture lacking enzyme extract were used as control. Then these tubes were placed under fluorescent lamp (30 W) for 10 min, lamp was turned off and absorbance of mixture was recorded at 560 nm using a spectrophotometer (IRMECO U2020). One unit of enzyme was taken as the amount of enzyme used to cause 50% inhibition in the photochemical reduction of NBT.

##### Catalase (CAT) and peroxidase (POD)

The activities of catalase (CAT) and peroxidase (POD) were evaluated according to the procedure given by Chance and Maehly (1955) with some minor modifications. The reaction mixture (3 ml) for CAT contains 5.9 mM H_2_O_2_, 50 mM potassium phosphate buffer (pH 7.8). To initiate the reaction 0.1 ml of the enzyme extract was added the above prepared mixture. The decrease in absorbance was read at 240 nm at every 20 s interval. One unit of CAT was taken as absorbance change of 0.01 units per min. The POD reaction mixture (3 ml) contained 40 mM H_2_O_2_, 20 mM guaiacol, 50 mM potassium phosphate buffer (pH 7.8) and 100 μl of enzyme extract. Then the change in absorbance at 470 nm was monitored after every 20 s. One unit of POD activity was defined as the change of 0.01 absorbance unit per min per mg of protein.

### Determination of Organic Compatible Solutes

#### Proline Determination

Free proline content was determined by using the protocol described by Bates et al. (1973). The third leaf from the top (0.25 g) was homogenized in 5 ml of 3% aqueous sulfosalicylic acid and homogenate was filtered through Whatman No. 2 filter paper. One ml of filtrate was taken and mixed with 1 ml of acid ninhydrin (1.25 g ninhydrin in 30 ml glacial acetic acid) and 1 ml of glacial acetic acid in a test tube. The mixture was vortexed shortly and heated at 100°C in a water bath for 1 h and then the reaction was terminated in the ice bath. 2 ml of toluene was added to the solution and vortexed for 15–20 s while it cooled. The chromophore containing proline was extracted from the aqueous phase in a test tube and warmed to laboratory temperature. The absorbance was taken at 520 nm using spectrophotometer (U2020 IRMECO).

#### Glycine Betaine

Grieve and Grattan (1983) method was followed for determination of glycine betaine content in leaf tissues. Briefly, 0.25 g dry material was homogenized with 5 ml of 0.5% toluene solution. Extract was centrifuged. 1 ml extract and 1 ml of 2N H_2_SO_4_ was mixed and 0.5 ml of this extract was taken in a separate test tube. 200 μL potassium tri-iodide was added in this extract and the test tube left in ice for 90 min. 2.8 ml distilled water was added then followed by addition of 6 ml 1,2-Dichloroethane. Upper layer was discarded and red lower layer was taken for reading at 365 nm.

### Nutrient Analysis of Plant Roots and Shoots

#### Digestion Method

To carry out plant mineral analysis, (which includes plant material digestion and mineral content determination) a method described by Allen et al. (1985) was followed, i.e., the dried (0.1 g) plant material was grinded well and placed in the 50ml flasks containingH_2_SO_4_. The mixture was boiled on a hot plate under a fume hood until digestion was completed which was indicated by the presence of white fumes in the flasks. Upon cooling, 50 ml distilled water was added and mixture was filtered by using Whatman paper # 42. Filtrate was further used for the determination of mineral nutrients.

#### Determination of Na^+^, K^+^, and Ca^2+^

Root and shoot sodium (Na^+^) potassium (K^+^) and calcium (Ca^2+^) were determined by using a flame photometer (Jenway, PFP-7, United Kingdom).

### Yield Parameters

At maturity, crop was harvested and data regarding spikes length per plant, number of spikelets per spike and 100 grains weight was recorded.

### Statistical Analysis

The data was analyzed using Statistix version. 8.1. An ANOVA (two-way) was performed to analyze the effect of treatments and errors associated with the experiment. Further, LSD (*p* = 0.05) test was used to identify significant difference among treatments means.

## Results

### Morphological Plant Attributes

**FIGURE 1:**
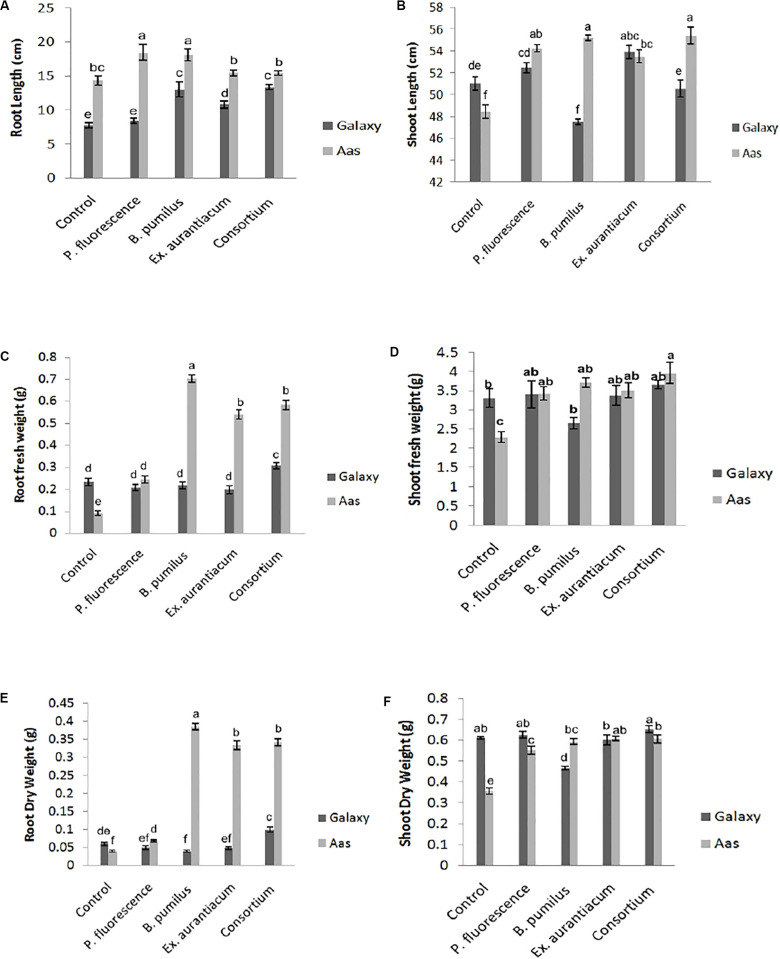
Effect of salt tolerant PGPR on growth attributes of two contrasting wheat varieties under saline condition. **(A)** Root length. **(B)** Shoot length. **(C)** Root fresh weight. **(D)** Shoot fresh weight. **(E)** Root dry weight. **(F)** Shoot dry weight.

Effects of different treatments (single strains and consortium) on root length were recorded on both wheat varieties. However, the salt tolerant variety; Aas-11, showed substantial increase compared to the salt sensitive variety; Galaxy-13. Seeds of variety Aas-11, bio-primed with *P. fluorescens* exhibited significant increase (28.59%) in root length followed by *B. pumilus* (26.22%) as compared to un-inoculated control plants. Plants inoculated with *E. aurantiacum* (T_4_) and consortium of PGPR (T_5_) expressed a non-significant increase in root length compared to untreated control plants. On the other hand, in variety Galaxy-13, all treatments exhibited significant increase except T_2_. The highest increase was recorded in T_5_ (71.72%) followed by T_3_ (67.35%) and T_4_ (39.33%), respectively (Figure 1A).

Shoot length was significantly increased upon inoculation with PGPR in variety Aas-11; consortium of PGPR (T_5_) represented maximum (14.36%) impact followed by T_3_ = 13.94%, T_2_ = 11.96% and T_4_ = 10.44%. In contrast to it, variety Galaxy-13, experienced variable effects on length of plant shoot like T_4_ (5.72%) followed by T_2_ (2.90%) exhibited maximum increase but T_5_ (consortium of PGPR) and T_3_ (*B. pumilus*) showed reduced shoot length than control pants (Figure 1B).

Root fresh and dry weights were also significantly increased among all treatments in the salt tolerant variety where T_3_ (0.704 and 0.386 g) showed highest increase followed by T_5_ (0.584 and 0.342 g), T_4_ (0.54 and 0.334 g) and T_2_ (0.244 and 0.068 g), respectively, as compared to un-inoculated control plants (T_1_ = 0.092 and 0.04 g). However, in Galaxy-13, only the combined application of PGPR manifested a significant increase in root fresh (30.72%) and dry (66.66%) weight than T_1_ (control). All other treatments showed decreased values ranging between (6 to 15%) except T_3_ (*B. pumilus*) where a slight increase was observed in root fresh weight. On the other hand, root dry weight was decreased in following manner T_2_ > T_4_ > T_3_, in contrast with control plants (Figures 1C,E).

Just like root fresh weight, PGPR inoculation imposed a significant positive impact on shoot fresh weight in Aas-11. T_5_ (72.73%) expressed highest values for shoot fresh weight followed by T_3_ (*B. pumilus* = 61.84%) > T_4_ (*E. aurantiacum* = 52.96%) > T_2_. (*P. fluorescens* = 49.39%) In the case of Galaxy-13, none of the treatments showed a significant increase but T_5_ (consortium of PGPR = 10.36%) showed maximum increase followed by T_2_ (2.83%) and T_4_ (1.80%) as compared to untreated control plants while T_3_ (19.95% decrease) showed the least value (Figure 1D).

Shoot dry weight was significantly increased among all PGPR treated plants such as T_4_ (*E. aurantiacum* = 70.39%) followed by T_5_ (PGPR consortium = 69.83%), T_3_ (*B. pumilus* = 65.92%) and T_4_ = 54.74% in salt tolerant variety (Aas-11) but in case of salt sensitive variety, this improvement was observed in T_5_ (6.86%) followed by T_2_ (2.61%) then control. However, T_4_ (*E. aurantiacum*) treated plants exhibited reduced value for shoot dry weight. *B. pumilus* (T_3_) inoculated plants showed significant reduction in value (Figure 1F). The variations in dry and fresh weight of different treatments depend on many physiological and environmental conditions.

### Physiological Plant Attributes

#### Water Relations in Plant

Water and osmotic potential were significantly improved in the salt tolerant variety (Aas-11). Maximum improvement was observed in plants inoculated with *E. aurantiacum* (T_4_ = 29.43% decrease). Other treatments represented the improved trend (19.52 to 10.36%) as follows; T_3_ > T_5_ and T_2_ in contrast with un-treated control plants. However, turgor potential was significantly lower among plants which received *P. fluorescens* (T_2_) as inoculation with gradual increase in T_5_, T_3_, and T_4_ as compared with un-inoculated plants. In the case of the salt sensitive variety (Galaxy-13), water and osmotic potential of PGPR treated plants were significantly improved except T_5_ (6.84%) which showed non-significant improvement in osmotic potential. T_2_ (*P. fluorescens*) treated plants exhibited the highest (22.09%) improved water potential followed by T_4_ (20.82%), T_3_ (18.925%), and T_5_ (13.85%) compared to control. whereas osmotic potential was highly improved in T_2_ followed by T_3_ (*B. pumilus*) and T_4_ (*E. aurantiacum*), respectively. Turgor potential was maximum in T_5_ (231.01%) followed by T_4_ while T_3_ (141 to 43%) results were at par with the control value. However, T_2_ showed a slight decrease compared to control (Table 2).

**TABLE 2:** Mean values of physiological attributes of two contrasting wheat genotypes inoculated with salt tolerant PGPR under saline condition.

Galaxy-13 variety	WP	OP	TP
T_1_	−1.57^a^	−1.62^a^	0.04^cd^
T_2_	−1.23^de^	−1.26^bc^	0.02^d^
T_3_	−1.28^bcd^	−1.33^b^	0.05^cd^
T_4_	−1.25^cd^	−1.36^b^	0.11^ab^
T_5_	−1.36^bc^	−1.51^a^	0.15^a^
**Aas-11 variety**			
T_1_	−1.38^b^	−1.54^a^	0.158^a^
T_2_	−1.24^d^	−1.33^b^	0.092^bc^
T_3_	−1.11^ef^	−1.17^cd^	0.059^cd^
T_4_	−1.06^f^	−1.08^d^	0.026^d^
T_5_	−1.12^ef^	−1.1^cd^	0.068^cd^

*WP, water potential (−MPa); OP, osmotic potential (−MPa), TP, turgor potential (MPa), T_1_, control; T_2_, P. fluorescens; T_3_, B. pumilus; T_4_, E. aurantiacum; T_5_, consortium. Different letters followed by mean values are significant (p < 0.05). Values with same letters are non-significant (p = 0.05).*

### Biochemical Plant Attributes

#### Accumulation of Osmotically Active Metabolites

**FIGURE 2:**
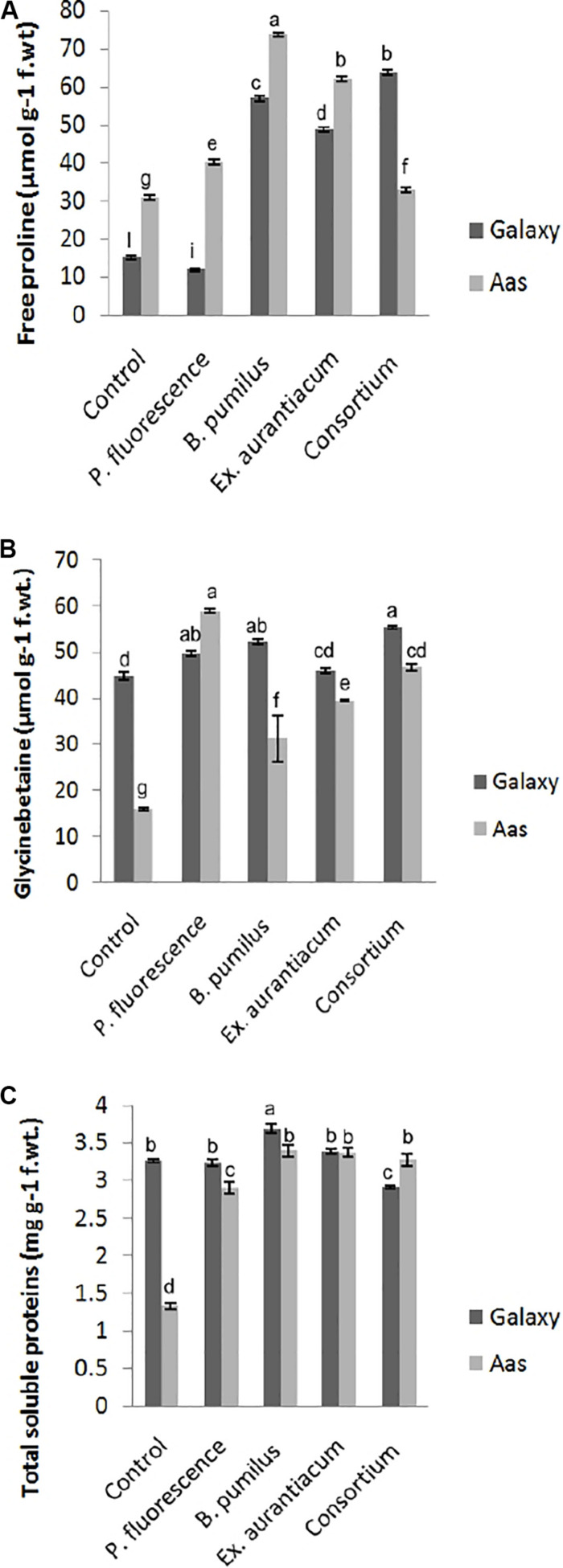
Effect of salt tolerant PGPR on accumulation of osmotically active metabolites of two contrasting wheat varieties under saline conditions. **(A)** Free proline content. **(B)** Glycine betaine content. **(C)** Total soluble proteins content.

Information about the effects of PGPR application on different biochemical attributes is presented in Figure 2. PGPR application caused a significant impact on the accumulation of free proline, glycine betaine and total soluble contents in the salt tolerant variety, i.e., Aas-11. However, effect of *E. aurantiacum* (T_4_) on glycine betaine accumulation remained non-significant. Free proline content was observed to be maximum among *B. pumilus* (T_3_ = 73.78 μmol g^–1^) treated plants followed by *E. aurantiacum* (T_4_ = 62.29 μmol g^–1^), *P. fluorescens* (T_2_ = 40.25 μmol g^–1^) and PGPR consortium (T_5_ = 32.85 μmol g^–1^), respectively (Figure 2A). The highest increase in glycine betaine content was recorded in T_5_ (55.21 μmol g^–1^) plants followed by T_3_ (52.06 μmol g^–1^) and T_2_ (49.48 μmol g^–1^) compared to the non-treated control (44.66 μmol g^–1^) plants (Figure 2B). Moreover, the total soluble proteins were increased in T_3_ (3.39 *unit/*mg protein) followed by T_4_ (3.37 *unit/*mg protein), T_5_ (3.27 *unit/*mg protein) and T_2_ (2.89 *unit/*mg protein) as compared to un-inoculated control (1.32 *unit/*mg protein) plants (Figure 2C). Examining the salt sensitive variety, i.e., Galaxy-13, free proline content was significantly enhanced in all treatments such as T_5_, T_3_, and T_4_ (63.81–48.89 μmol g^–1^) but T_2_ treated plants represented a decrease in its level. However, it differed non-significantly to the control plants. While the accumulation of glycine betaine was significantly high in T_5_ (63.81 μmol g^–1^) followed by T_3_, T_2_, and T_4_ which ranged between 52.06 and 45.9 μmol g^–1^, respectively (Figure 2B). However, only *B. pumilus* (T_3_ = 3.68 *unit/*mg protein) manipulated a significant increase in total soluble proteins content in contrast with un-treated control plants while the rest of the treatments showed a non-significant increase (10.69 to 0.78%) in their content which is as follow, T_5_ > T_4_ and T_2_ (Figure 2C).

#### Determination of Antioxidant Enzymatic Activity

**FIGURE 3:**
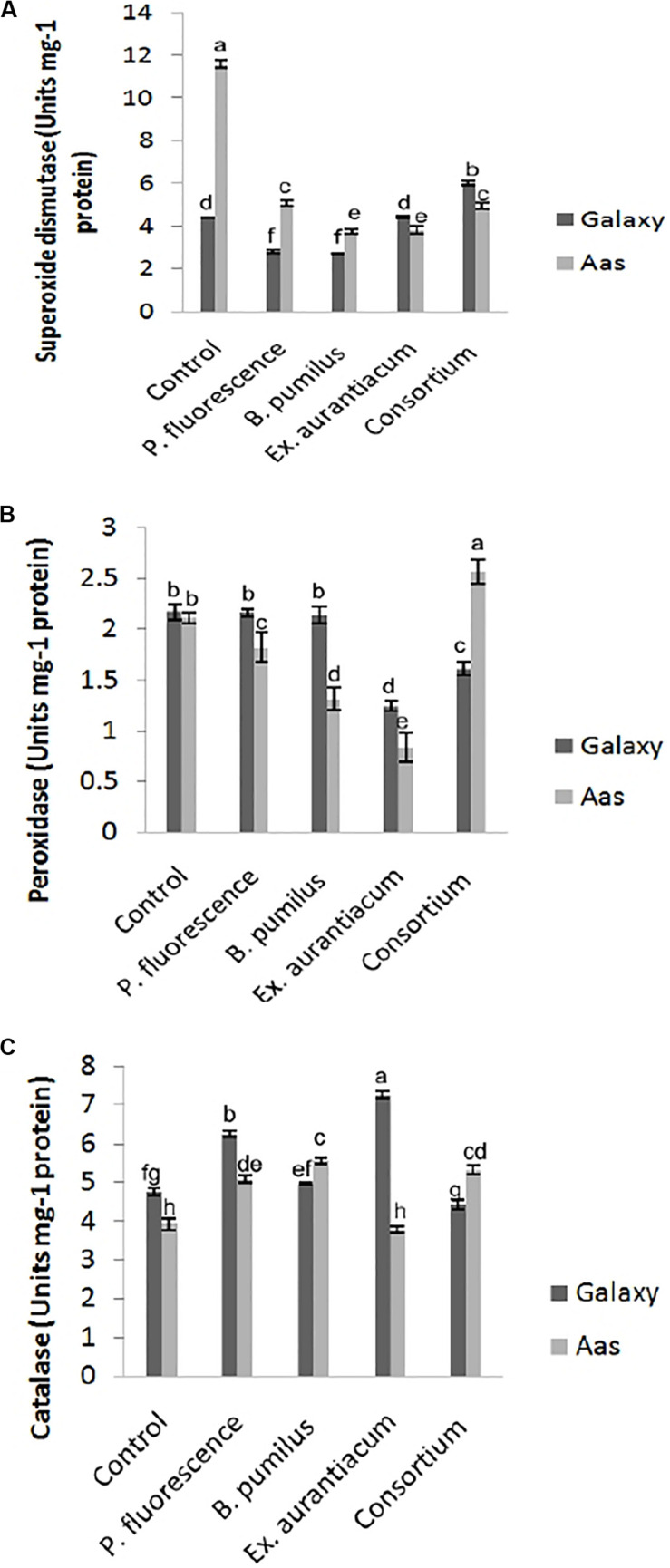
Effect of salt tolerant PGPR on the activity of enzymatic antioxidants of two contrasting wheat varieties under saline conditions. **(A)** Superoxide dismutase activity. **(B)** Peroxidase activity. **(C)** Catalase activity.

All treatments imposed their substantial impact on activities of antioxidant enzymes in the salt tolerant variety (Figure 3). Significant reduction in activity of superoxide dismutase was observed among all treatments (Figure 3A). The least activity was recorded in T_3_ (*B. pumilus* = 3.744 *unit/*mg protein) with gradual increase in T_4_ (*E. aurantiacum* = 3.834 *unit/*mg protein) < T_5_ (Consortium = 4.958 *unit/*mg protein) < T_2_ (*P. fluorescens* = 5.070 *unit/*mg protein). A variable effect on the activity of peroxidase was observed among all PGPR treated plants. (Figure 3B) i.e., least activity was observed in T_4_ (0.834 *unit/*mg protein) with gradual increase among T_3_ < T_2_ (1.314 and 1.826 *unit/*mg protein) which is lower than control (T_1_ = 2.113 *unit/*mg protein) plants. However, peroxidase activity in T_5_ (PGPR consortium = 2.574 *unit/*mg protein) inoculated plants, significantly higher than un-inoculated control. Catalase activity was non-significantly increased in T_3_ > T_5_ > T_2_. However, plants treated with *E. aurantiacum* (T_4_ = 3.799 *unit/*mg protein) expressed reduced activity in comparison with un-inoculated control plants (Figure 3C).

On the other hand, in the case of the salt sensitive variety (Galaxy-13), a significant decrease in superoxide dismutase activity was recorded in *B. pumilus* (T_3_ = 2.695 *unit/*mg protein) with a gradual increase in *P. fluorescens* (T_2_ = 2.79 *unit/*mg protein) while no change was observed in *E. aurantiacum* (T_4_ = 4.42 *unit/*mg protein) treated plants (Figure 3A). Enhanced activity was found in only T_5_ (PGPR consortium = 6.040 *unit/*mg protein) treated plants. Peroxidase activity was significantly lower in T_4_ (1.242 *unit/*mg protein) treated with gradual increase in T_5_ (1.609 *unit/*mg protein) plant leaves which differed significantly with other treatments. Non-significant reduction in peroxidase enzyme activity was observed among T_2_ > T_3_ as compared with control plants (Figure 3B). Catalase activity was significantly enhanced among *E. aurantiacum* inoculated plants T_4_ = 7.262 *unit/*mg protein followed by T_2_ = 6.24 *unit/*mg protein treated plants while T_3_ treated plants exhibited non-significant increase in its value. However, T_5_ plants, whose seeds were bioprimed with PGPR consortium, showed 6.86% reduced activity compared to control plants but the difference was not significant (Figure 3C).

### Analysis of Mineral Nutrients in Plant Roots and Shoots

Variable results on nutrient acquisition were recorded upon PGPR application among both varieties (Table 3). Sodium content in plant roots remained non-significant in all treatments of the salt tolerant variety (Aas-11). All treatments showed a slightly reduced sodium uptake compared to the control except the T_5_ (PGPR consortium) treated plant, which showed no change in its value. On the other hand, in the salt sensitive variety (Galaxy-13), a slight increase (11 and 29%) in its acquisition was observed in T_2_ (*P. fluorescens*) and T_4_ (*E. aurantiacum*) inoculated plants while the effect of rest of the treatments remained un-changed as compared to control plants.

**TABLE 3:** Mean values of mineral nutritional attributes of two contrasting wheat genotypes inoculated with salt tolerant PGPR under saline condition.

Galaxy-13 variety	R Na^+^	S Na^+^	R K^+^	S K^+^	R Ca^2+^	S Ca^2+^
T_1_	0.60^a^	9.8^de^	3.37^de^	2.60^de^	0.6^a^	2.25^c^
T_2_	0.90^a^	12.5^b^	4.70^c^	3.20^cd^	0.9^a^	2.20^c^
T_3_	0.60^a^	9.0^ef^	4.50^cd^	2.10^e^	0.6^a^	1.4^d^
T_4_	0.80^a^	12.4^b^	4.50^cd^	2.60^de^	0.8^a^	2.62^c^
T_5_	0.60^a^	11.4^bc^	7.20^b^	3.00^cd^	0.6^a^	1.40^d^
**Aas-11 variety**						
T_1_	0.80^a^	7.6^f^	2.20^e^	3.70^bc^	0.8^a^	0.90^d^
T_2_	0.70^a^	14.6^a^	4.37^cd^	4.30^ab^	0.7^a^	3.50^b^
T_3_	0.70^a^	12.1^b^	6.50^b^	3.30^cd^	0.7^a^	3.75^*b*^
T_4_	0.70^a^	10.6^cd^	8.50^a^	4.5^a^	0.7^a^	4.50^a^
T_5_	0.80^a^	12.8^b^	6.00^b^	4.80^a^	0.8^a^	4.50^a^

*R Na^+^, root sodium (mg g^–1^ dry wt.); S Na^+^, shoot sodium (mg g^–1^ dry wt.); R K^+^, root potassium (mg g^–1^ dry wt.); S K^+^, shoot potassium (mg g^–1^ dry wt.); R Ca^2+^, root calcium (mg g^–1^ dry wt.); S Ca^2+^, shoot calcium (mg g^–1^ dry wt.); T_1_, control; T_2_, P. fluorescens; T_3_, B. pumilus; T_4_, E. aurantiacum; T_5_, consortium. Different letters followed by mean values are significant (p < 0.05). Values with same letters are non-significant (p < 0.05).*

Shoot sodium acquisition was significantly increased among both varieties upon PGPR inoculation. Where T_2_ showed maximum sodium uptake followed by T_5_, T_3_, and T_4_. Similarly, significant results were recorded in Galaxy-13, where the maximum value was represented in T_2_ followed by T_4_ and T_5_ plants. However, T_3_ (*B. pumilus*) showed slight low (8.16%) sodium uptake compared to control.

In variety, Aas-11, maximum potassium content in root tissues was recorded in T_4_ (286.36%) followed by T_3_ > T_5_ > T_2_ (195.45–98.28%) treated plants. On the other hand, highest amount of potassium in plant shoots was found in T_5_ (29.72%) followed by T_4_ (21.62%) and T_2_ (16.21%) as compared with un-treated control plants. Collectively, root and shoot potassium uptake was significantly increased in the salt tolerant variety upon PGPR inoculation except T_3_ which exhibited only a slight decrease in shoot potassium content compared to the control plants. In the case of salt susceptible variety, T_5_ (PGPR consortium = 113.3%) followed by T_2_ (*P. fluorescens*) treated plants exhibit significant increase in root potassium content while this increase remained non-significant in T_4_ and T_3_ treated plants. Variable impact on potassium uptake by plant shoots was observed among all treatments where T_2_ (23.07%) followed by T_5_ (15.38%) showed a non-significant increase in value. While its content among *E. aurantiacum* inoculated plants remained similar to control, however, T_3_ exhibited slight reduced potassium value.

PGPR imposed non-significant impact on root calcium content in both varieties. PGPR consortium showed similar values to control but rest of treatments exhibited slight (12.5%) decrease in root calcium pool in salt tolerant variety. However, in the salt sensitive variety, maximum root calcium was recorded in T_2_ (50%) followed by T_4_ (33%) treated plants than control but the rest of the two treatments showed no significant change in its value as compared with un-treated control plants.

Calcium content in plant shoots was substantially increased among all treatments in Aas-11 where values for T_5_ and T_4_ were equal (400% increase) and followed by T_3_ and T_2_ (316 and 288%). On the other hand, in Galaxy-13, only T_5_ and T_3_ (37.78% increase) treated plants showed a significant increase in calcium content while T_4_ was at par with control plants. However, T_2_ showed a slight decrease in its value compared to the control.

### Yield Attributes of Plant

Yield contributing components including spike length, number of spikelets per spike and 100 grains weight were substantially increased upon PGPR seed inoculation either as single strain or consortium (Table 4). Maximum values were recorded in plants treated with *B. pumilus* followed by PGPR consortium (T_5_) and *E. aurantiacum* (T_4_) in salt tolerant variety. However, impact of *P. fluorescens* inoculation was only evident in increasing 100 grains weight. Whereas in salt susceptible variety; *Galaxy-13, P. fluorescens* inoculation exhibited maximum values among all yield contributing components, followed by T_5_ and T_4_. While the effect of *B. pumilus* inoculation was at par with un-inoculated control plants.

**TABLE 4:** Mean values of yield attributes of two contrasting wheat genotypes inoculated with halophilic PGPR under saline condition.

Galaxy-13 variety	Spike length	No. of spikelets spike^–1^	100 G. wt
T_1_	6.84^f^	21.4^d^	3.46^d^
T_2_	12.72^ab^	37.8^ab^	4.6^bc^
T_3_	7.64^ef^	24.6^cd^	4.52^c^
T_4_	9.30^d^	27.8**^C^**	5.16^ab^
T_5_	11.02^c^	34.8^b^	5.30^a^
**Aas-11 variety**			
T_1_	8.28^de^	25.8**^C^**	3.56^d^
T_2_	8.72^de^	26.8**^C^**	4.38^c^
T_3_	13.60^a^	40.2^a^	5.46^a^
T_4_	12.28^b^	34.8^b^	5.24^a^
T_5_	13.46^ab^	39.8^a^	5.28^a^

*Spike length (cm), No. of spikelets spike^–1^; 100 G. wt, 100 grains weight (g); T_1_, control; T_2_, P. fluorescens; T_3_, B. pumilus; T_4_, E. aurantiacum; T_5_, consortium. Different letters followed by mean values are significant (p < 0.05). Values with same letters are non-significant (p < 0.05).*

## Discussion

Soil salinity is a prevalent environmental restraint to agriculture productivity and food security. Salt stress is responsible for 20–50% yield losses of important agricultural commodities including wheat, rice and maize around the world (Subiramani et al., 2020). So, there is a pressing need to adapt new sustainable approaches in addition to the use of organic or inorganic soil amendments along with salt resistant crop varieties to improve the productivity of such problematic soils (Egamberdieva et al., 2019). Exploitation of salt tolerant PGPR has recently emerged as an effective strategy to handle aforesaid situation (Grover et al., 2011). These halophilic and halotolerant microorganisms are capable of being sustained in hyper-saline habitats (Talaat, 2018). Main mechanisms responsible for their survival under stressful environment include *de novo* synthesis or uptake of osmoprotectants and specialized ion transport systems like Na^+^/H^+^ antiporters, respectively (Egamberdieva et al., 2019). Bacterial species belonging to genera Pseudomonas, *Bacillus, Enterobacter, Agrobacterium, Streptomyces, Klebsiella*, and *Ochromobacter* have been extensively reported as efficient bio-inoculants in saline agriculture (Sharma et al., 2016; Singh and Jha, 2016; Sarkar et al., 2018).

The main focus of this study was to investigate the potential impact of salt tolerant PGPR on wheat growth and yield enhancement through modulation of morpho-physiological and biochemical mechanisms. For this purpose, three PGPR strains were used. A strain of *P. fluorescens* was isolated from roots of maize plant grown in non-saline habitat. The other two microbes, *B. pumilus* and *E*. *aurantiacum* were isolated from roots of wheat, grown in Khewra salt range (Ullah, 2019). The salt tolerant potential of the latter two PGPR strains was higher than the first (Ullah, 2019).

Previous studies (Ansari et al., 2019; Xie et al., 2019) have demonstrated the significant contribution of several bacterial sp. belonging to *Pseudomonas*, *Bacillus*, and *Exiguobacterium* (Kasana and Pandey, 2018) genera in plant growth promotion under growth limiting conditions.

Safari et al. (2018) reported the enhanced salt tolerance index and substantial increase in germination percentage and seedling vigor in wheat upon inoculation with *P. fluorescens* under NaCl induced salinity. A similar response on barley growth and yield (spike length, fertility index and grains weight) parameters was described by Azadikhah et al. (2019) when treated with ACC deaminase producing *P. fluorescens* under salt stress. Nadeem et al. (2016) claimed that plant growth promotion via treatment with ACCD, IAA, and siderophore producing *P. fluorescens* is directly related to its better colonizing ability in plant rhizosphere. A number of studies on the potential of *Bacillus* sp. have also been documented in literature. Din et al. (2019) reported that bacterial strains belonging to *Bacillus* genus showed substantial role in salt stress alleviation in wheat due to their capability to produce EPS, ACCD and IAA production *in vitro*. Xie et al. (2019) and Ansari et al. (2019) documented that the supportive role in salt stress alleviation and wheat growth improvement revealed by *B. pumilus* inoculation was related to escalated levels of photosynthesis, transpiration and proline accumulation as well as reduced antioxidant levels. Bacillus strains resistant to salt stress contributed to improve K^+^ and Ca^2+^ acquisition and enhancement of protein and nitrogen content in rice seedlings grown under salt stress (Khattak et al., 2019). However, only limited data is available regarding the PGP activities showed by *E*. *aurantiacum* (Ullah and Bano, 2019). Strahsburger et al. (2018) reported the draft genome of *E*. *aurantiacum* strain PN47, data obtained from this study confirmed its adaptive features in hyper-osmotic and alkaline environment.

Our findings are concomitant with the previously reported literature that the application of salt tolerant PGPR strains *P. fluorescens*, *B. pumilus*, and *E*. *aurantiacum* substantially increased the growth and yield of wheat crop grown under saline conditions. It may be due to the fact that these PGPR were able to metabolize ACC deaminase, solubilize insoluble phosphate minerals and produce a significant quantity of IAA (Ullah and Bano, 2019). However, in our study, *B. pumilus* (T_3_) and *E*. *aurantiacum* (T_4_) showed promising results in the case of the salt tolerant variety; Aas-11 while the salt susceptible variety; Galaxy-13 performed more effectively upon inoculation with *P. fluorescens* (T_2_) and synergetic behavior of inoculated PGPR strains was quiet eminent too (T_5_). This variant response of inocula can be regarded as the PGPR colonization potential in rhizosphere varies with plant genotype, species, and developmental stage etc. (Delaplace et al., 2015; Poli et al., 2016; Wintermans et al., 2016) as well as physio-chemical characteristics of surrounding soil and nutrition competition among microbial communities. Basically, phytomicrobiome development is recruited by the plant itself which excretes various type of root exudates (Chaparro et al., 2012; Trabelsi and Mhamdi, 2013) and this interaction is regulated at biochemical and genetic level through signal transduction (Nelson and Sadowsky, 2015; Massalha et al., 2017; Smith et al., 2017).

In the current experiment, *B. pumilus* (characterized for *in vitro* PGP potential) inoculated plants (variety; Aas-11) showed maximum root fresh and dry weight, accumulation of free proline and total soluble proteins contents in leaf tissues along with reduced activity of enzymatic antioxidants activity. However, reduced activity of antioxidant is an evident feature of PGPR induced modulation of plant physiology that resulted in reduced ROS contents (Singh et al., 2016). *E*. *aurantiacum* showed the highest shoot dry weight and improved water and osmotic potential. Maximum Ion (K^+^ and Ca^2+^) acquisition by root tissues and decreased level of shoot sodium content suggest the efficacy of this PGPR strain in regulation of plant ion transporters to inhibit sodium uptake and promotion of potassium and calcium uptake by plant (Strahsburger et al., 2018). Combined application of PGPR (T_5_) exhibited an increase in shoot length, fresh weight, K^+^ and Ca^2+^ amount and glycine betaine content. Here, the synergetic role manipulated by PGPR was quite evident in the mitigation of ionic toxicity upon exposure to salt stress and resulted in increased growth and yield of wheat plants. A significant difference in shoot length and turgor potential was noted in *P. fluorescens* treated plants as compared to un-inoculated control plants.

On the other hand, if we look at salt sensitive variety; Galaxy-13, the effect of *P. fluorescens* on improved water relations, osmolyte (glycine betaine) accumulation, reduced activity of superoxide dismutase and elevated levels of shoot potassium and root calcium contents were recorded as compared to control plants. The implication of PGPR consortium (T_5_) showed improved root/shoot growth parameter, increased root potassium content and maximum amount of proline content accumulation. While shoot length and calcium content were maximum in *E*. *aurantiacum* treated plants in comparison with un-treated control plants. Whereas total soluble protein content was highest in *B. pumilus* inoculated plant tissues.

The above-mentioned outcomes revealed the supportive role of inoculated PGPR strains in growth and yield enhancement of wheat crop under salt stressed conditions. It also suggests that IAA producing bacteria accelerated the modulation of the plant’s morpho(growth parameters)-physiological (water relations) characteristics (Emami et al., 2019) and biochemical (osmolytes accumulation and reduced activity of enzymatic antioxidants) attributes. Moreover, inoculation assisted the plant to maintain nutrition balance via increased K^+^ and Ca^2+^ uptake and reduced sodium ion acquisition. Hence, PGPR improved the yield of wheat crop planted under stressful conditions.

These results are consistent with the previous reports which showed increased plant growth and yield upon inoculation with salt tolerant PGPR under salt stress (Singh and Jha, 2016). Thus, PGPR strains, *P. fluorescens*, *B. pumilus* and *E*. *aurantiacum* can be regarded as promising microorganisms to formulate biofertilizer specific for saline soils to minimize wheat crop yield penalties caused by soil salinization. Many PGPR strains belonging to genera *Pseudomonas, Ochrobactrum, Bacillus, Azospirillum, Azotobacter, Rhizobium, Stenotrophomonas, Serratia*, and *Enterobacteria* (Maleki et al., 2011) have been extensively reported and exploited as effective candidates to be formulated as biofertilizers – green biotechnology, as an alternative sustainable approach in saline agriculture (Egamberdieva et al., 2019).

## Conclusion

Outcomes of the present study inferred that PGPR employed beneficial impact on physio-chemical attributes of inoculated plants consequently leading toward alleviation of salinity induced damages through improved water relations, enhanced compatible solutes accumulation, stimulated potassium and calcium acquisition and reduced antioxidant enzymes activity. These alterations in cellular metabolism ultimately led to the improved growth and yield among both salt tolerant and susceptible varieties under salt stress. However, the salt tolerant variety showed far better growth and yield than the sensitive variety. *B. pumilus* and *E. Aurantiacum* single strains and consortium manifested a more evident impact on the salt resistant genotype while in the case of the salt sensitive genotype, *P. fluorescens* single strain and consortium played a pivotal role in growth and yield improvement. Further experimentation at multiple field locations and detailed investigation of molecular mechanisms in near future can lead toward application of these microbes as biofertilizer in salt affected soil for enhanced wheat crop production.

## Data Availability Statement

All datasets presented in this study are included in the article/**Supplementary Material**.

## Author Contributions

AN conducted the experiments and sampling. AN and MS designed the project. AN, MS, and A carried out data analysis and manuscript preparation. MS, A, FM, AI, MI, and MM reviewed and edited the manuscript. All authors contributed to the article and approved the submitted version.

## Conflict of Interest

The authors declare that the research was conducted in the absence of any commercial or financial relationships that could be construed as a potential conflict of interest. The reviewer MN declared a shared affiliation with the authors to the handling Editor at the time of review.
